# 541例女性晚期非小细胞肺癌患者的预后因素分析

**DOI:** 10.3779/j.issn.1009-3419.2011.03.21

**Published:** 2011-03-20

**Authors:** 梅娜 吴, 玉艳 王, 彤同 安, 军 赵, 鹭 杨, 建春 段, 志杰 王, 明磊 卓, 桦 白, 叙仪 刘, 洁 王

**Affiliations:** 100142 北京，北京大学临床肿瘤学院，北京肿瘤医院胸部肿瘤内科 Department of Thoracic Oncology, Beijing Cancer Hospital, Beijing University Oncology College, Beijing 100142, China

**Keywords:** 肺肿瘤, 女性, 生存, 预后, Lung neoplams, Female, Survival, Prognosis

## Abstract

**背景与目的:**

随着女性肺癌发病率的攀升，其独特的临床和流行病学特征及良好预后引起了学界的关注。本研究通过回顾性分析女性晚期非小细胞肺癌（non-small cell lung cancer, NSCLC）患者的临床资料，探讨其预后相关因素。

**方法:**

收集541例女性晚期NSCLC患者的临床资料，并随访至死亡。主要观察指标为总生存（overall survival, OS）。采用SPSS 11.0统计软件进行生存分析。

**结果:**

全组腺癌占80.2%（434/541），总体中位OS为15个月（95%CI: 13.87-16.13），1年、2年、5年生存率分别为58.8%、23.7%和3.20%。单因素分析显示，临床分期、ECOG评分、体重下降、临床症状、血行转移和一线治疗后化疗方案数>1、一线化疗有效、曾接受靶向治疗或放疗均与中位OS明显相关（*P*值均 < 0.05）。治疗前体重下降、ECOG评分、靶向治疗及一线化疗有效为生存的独立预后因素（*P*值均 < 0.05）。

**结论:**

女性晚期NSCLC患者的病理类型以腺癌为主，体重下降、ECOG评分、接受靶向治疗及一线化疗有效可能成为女性晚期NSCLC患者生存的独立预后指标。

## Abstract (en)

**Background and objective:**

As there is a sharp increase in the incidence of lung cancer in women in recent years, it has brought broad concerns with its unique clinical and epidemiological characteristics and better prognosis. The aim of this study is to analyze the clinical data of women with advanced non-small cell lung cancer (NSCLC) retrospectively to explore the prognostic factors.

**Methods:**

Clinical data of 541 female patients with advanced NSCLC were collected and followed up till death. The primary endpoint is overall survival (OS). SPSS 11.0 statistical analysis software was used for univariate and multivariate analysis.

**Results:**

The mean age is 59 years (20 years-86 years), adenocarcinoma account for 80.2% (434/541). The median OS was 15 months (95%CI: 13.87-16.13), and 1, 2, 5-year survival rates were 58.8%, 23.7% and 3.20% respectively. Univariate analysis showed that clinical stage, ECOG score, weight loss, clinical symptoms, liver/bone/brain metastasis and received more than one chemotherapy regimen, good response to the first-line chemotherapy, EGFR-TKI targeted therapy and radiotherapy treatment were significantly correlated with the OS and survival rate (*P* < 0.05). Combined with multivariate analysis, weight loss before treatment, ECOG score, received EGFR-TKI targeted therapy and response to first-line chemotherapy were independent prognostic factor for survival (*P* < 0.05).

**Conclusion:**

There is a higher percentage of adenocarcinoma in female NSCLC. Weight loss before treatment, ECOG score, EGFR-TKI targeted therapy and response to first-line chemotherapy may become independent prognostic factors for survival of female patients with advanced NSCLC.

20世纪初关于女性肺癌的报道还很少见，但流行病学研究发现1990年-2003年美国女性肺癌新发病例数增加了60%，而男性则保持稳定，肺癌已经成为女性恶性肿瘤的第一位死亡原因^[1]^。我国卫生部全国肿瘤防治研究办公室提供的资料^[2]^也显示2000年-2005年间中国女性肺癌新患者从12万人增至17万人，增长速度已超过男性。因此提高对女性肺癌的关注已经成为改善肺癌总体生存的重要因素。

目前研究已经发现，女性肺癌在流行病学、临床病理特征、分子标志物、治疗反应以及预后预测因素方面有别于男性肺癌，具有其相对独特的表现。与男性肺癌相比，女性更易发生腺癌和小细胞肺癌，而少见鳞状细胞癌。女性肺癌诊断年龄偏早，常无明确吸烟史，确诊肺癌时的分期较早，且任何分期的患者生存期都较男性明显延长。就各期肺癌总体而言，女性5年生存率为15.6%，男性为12.5%^[3]^。欧洲肺癌协作组织的多因素分析提示女性的相对死亡风险为0.7（*P*=0.003）^[4]^。O’Connell等^[5]^的研究也证明女性中位生存时间为12.4个月，男性为8.8个月（*P*=0.001)。以上均体现出女性肺癌具有明显的生存优势，也预示着性别分层分析对肺癌临床研究具有举足轻重的作用。在女性NSCLC、特别是晚期患者中，到底哪些因素是决定预后的关键？这个问题目前尚无定论。

本研究通过回顾性分析北京肿瘤医院胸部肿瘤内科1995年9月-2007年12月收治的女性晚期非小细胞肺癌（non-small cell lung cancer, NSCLC）患者的临床特征和随访资料，探讨影响其生存的预后相关因素，以期为指导临床治疗决策、判断预后提供参考依据。

## 1 资料与方法

### 1.1 一般资料

1995年9月-2007年12月北京肿瘤医院胸部肿瘤内科（原呼吸肿瘤内科）收治的经组织或细胞学诊断的女性晚期NSCLC患者共541例，均接受过至少一次化疗。收集患者的临床资料，包括年龄、TNM分期、体力状况、病理类型、吸烟状况、临床症状、转移部位（脑、骨、肝）、接受治疗情况（化疗、靶向治疗）及疗效等，主要观察指标为患者的中位生存时间和总生存（overall survival, OS），由统计室及本科室进行电话随访至2009年12月底，失访率为7.2%（39例）。

### 1.2 统计分析

应用SPSS 11.0统计分析软件，生存分析采用*Kaplan-Meier*法并进行*Log-rank*时序检验。通过*COX*模型进行生存的多因素分析。*P* < 0.05为有统计学差异。

## 2 结果

1995年9月-2007年12月收治的经组织或细胞学确诊的女性晚期NSCLC患者1, 683例，同期接受过至少一次化疗者541例，占32.14%。截至末次随访时间，全组患者的1年、2年和5年生存率分别为58.8%（325/541）、23.7%（120/541）和3.2%（8/541），中位OS为15个月（95%CI: 13.87-16.13）。

### 2.1 治疗前状态与生存

#### 2.1.1 年龄

全组平均年龄为59岁（20岁-86岁）。≥70岁的患者占15.1%（82/541）， < 70岁的患者占84.9%（459/541），中位生存时间分别为17个月（95%CI: 13.86-20.14）和15个月（95%CI: 13.84-16.16），两组之间差异无统计学意义（*P*=0.063）。具体见表 1。

**1:** 女性晚期非小细胞肺癌患者预后因素与生存关系的单因素分析结果 Univariate analysis results of prognostic factors for female patients with advanced non-small cell lung cancer

Progniostic factor	Group	*n*	Mean OS(month)	1-yearsurvival rate (%)	2-yearsurvival rate (%)	*χ*^2^	*P*
Age (year)	< 70	459	15	58.4	23.3	3.46	0.063
	≥70	82	17	63.9	30.0		
Pathology	Adenocarcinoma	434	15	60.5	25.88	7.3	< 0.001
	Squamous cell carcinoma	67	14	56.80	22.46		
	Others	40	12	44.97	5.6		
Clinical stage	Ⅲb	61	17	73.77	29.51	24.96	< 0.001
	Ⅳa	65	22	76.92	44.62		
	Ⅳb	415	14	53.97	20.0		
ECOG PS	0-1	417	17	65.58	28.43	39.71	< 0.001
	≥2	124	10	36.58	8.9		
Weight loss	< 5%	373	17	63.97	26.79	13.37	< 0.001
	≥5%	168	12	47.09	17.30		
Symptom	None	83	22	70.44	40.57	20.20	< 0.001
	Chest	357	15	60.27	22.01		
	Other organ	101	12	44.58	16.31		
Smoking status	Non-Smoker	509	15	60.26	24.28	1.52	0.2180
	Smoker	32	12	37.22	18.56		
Brain metastasis	No	378	17	62.96	27.51	18.74	< 0.001
	Yes	163	12	49.60	15.70		
Bone metastasis	No	250	18	69.68	33.65	38.56	< 0.001
	Yes	291	12	49.46	15.45		
Liver metastasis	No	485	16	60.92	25.84	17.28	< 0.001
	Yes	56	10	41.07	6.6		
Lung metastasis	No	309	15	59.1	23.05	0.10	0.751, 9
	Yes	232	14	58.62	25.15		
Response of 1-line	Disease control	321	17	69.17	28.23	46.00	< 0.001
	Progression	185	11	38.82	8.57		
Chemotherapy regimen after 1-line	≥2	315	18	71.40	32.06	49.93	< 0.001
	1	226	10	40.87	12.40		
Radiotherapy	Ever	265	17	64.83	27.73	7.31	0.006, 9
	Never	276	14	52.97	20.84		
EGFR-TKI treatment	Ever use	160	23	84.01	40.52	42.29	< 0.001
	Never use	381	12	47.95	16.77		
OS: overall survival; ECOG: Eastern Cooperative Oncology Group; PS: performance status; EGFR-TKI: epidermal growth factor receptor tyrosinekinase inhibitor.							

#### 2.1.2 病理类型

腺癌占80.2%（434/541），鳞癌占12.4%（67/541），其它类型占7.4%（40/541），腺癌中位OS为15个月（95%CI: 13.8-16.2）长于鳞癌14个月（95%CI: 10.2-17.8），但差异无统计学意义（*P*=0.89）。腺癌较其它病理类型中位OS为12个月（95%CI: 10.5-13.1）及1年、2年生存率具有明显生存优势（*P*=0.03）。具体见表 1。

#### 2.1.3 临床分期

参照2009年国际肺癌研究学会推荐的TNM分期系统，Ⅲb期占11.3%（61/541），Ⅳa期占12.0%（65/541），Ⅳb期占76.7%（415/541）。Ⅳa期和Ⅳb期之间中位OS、1年生存率和2年生存率差异具有统计学意义（*P* < 0.01），具体见表 1。Ⅳa期患者中位OS较Ⅲb期延长（22个月*vs* 17个月），但差异无统计学意义（*P*=0.516）。

#### 2.1.4 治疗前体力状态评分

按照ECOG评分标准，0分-1分者占77.1%（417/541），ECOG 2分者占23.9%（124/541），前者中位生存时间、1年生存率和2年生存率均明显优于后者（*P* < 0.01）。具体见表 1。

#### 2.1.5 体重变化

治疗前体重无下降者占6 8. 9 %（373/541），体重下降者占31.1%（168/541）。两组之间中位生存时间、1年生存率和2年生存率差异具有统计学意义（*P*=0.000, 3）。具体见表 1。

#### 2.1.6 治疗前症状

体检发现（无症状）者占15.3%（8 3 / 5 4 1），以胸部症状为首发表现者占6 5. 9%（357/541），以转移症状就诊者占17.8%（101/541）。三组中位生存时间分别为22个月（95%CI: 17.0-27.0）、15个月（95%CI : 13.74-16.26）和12个月（95%CI : 10.43-13.57），1年生存率分别为70.44%、60.27%和44.58%，2年生存率分别为40.57%、22.01%和16.31%，无症状者与有症状者之间差异具有统计学意义（*P* < 0.01）。具体见表 1。

#### 2.1.7 吸烟

吸烟患者32例，占5.9%，与不吸烟患者相比，中位OS、1年生存率和2年生存率无统计学差异（*P*=0.22）。具体见表 1。

#### 2.1.8 转移情况

脑、骨、肝和对侧肺转移患者的发生比例分别为30.1%（163/541）、53.8%（291/541）、10.3%（56/541）和42.9%（232/541）。发生前三种远处转移对患者的中位OS、1年生存率和2年生存率上均具有明显影响（*P* < 0.01），而有无肺转移对生存的影响无明显性（*P*=0.75）。具体见表 1。

### 2.2 治疗情况与生存

541例患者均接受过至少1周期化疗，可评价疗效者506例，一线治疗总有效率为31%（157/506），疾病控制率为63.5%（321/506）。

#### 2.2.1 一线化疗疗效

一线化疗有效者与无效者中位OS分别为17个月（95%CI: 15.0-20.9）和11个月（95%CI: 10.9-13.1），1年生存率分别为69.17%和38.82%，2年生存率分别为28.23%和8.57%，差异具有统计学意义（*P* < 0.01）。

#### 2.2.2 接受二线以上方案治疗

仅接受1个方案化疗的患者226例（41.8%），与接受2种以上方案化疗的患者相比，中位OS、1年和2年生存率均明显降低（*P* < 0.01）。具体见表 1。

#### 2.2.3 靶向治疗

160例（30.0%）患者在治疗过程中曾接受酪氨酸激酶抑制（tyrosine kinase inhibitor, TKI）治疗，381例（70.0%）未接受。接受过靶向治疗者中位OS、1年生存率、2年生存率均明显优于未接受者（*P* < 0.01）。具体见表 1。

#### 2.2.4 放疗

51%（276/541）患者在治疗过程中曾接受姑息放射治疗，与未接受过放疗者比，中位OS、1年生存率和2年生存率差异均具有明显性（*P*=0.006, 9）。具体见表 1。

### 2.3 多因素预后分析

多因素分析显示女性晚期NSCLC患者的疗前体重下降（*P* < 0.01）、ECOG评分（*P*=0.003）、靶向治疗（*P* < 0.01）及一线化疗有效（*P* < 0.01）等为生存的独立预后因素。具体见图 1。

**1:**
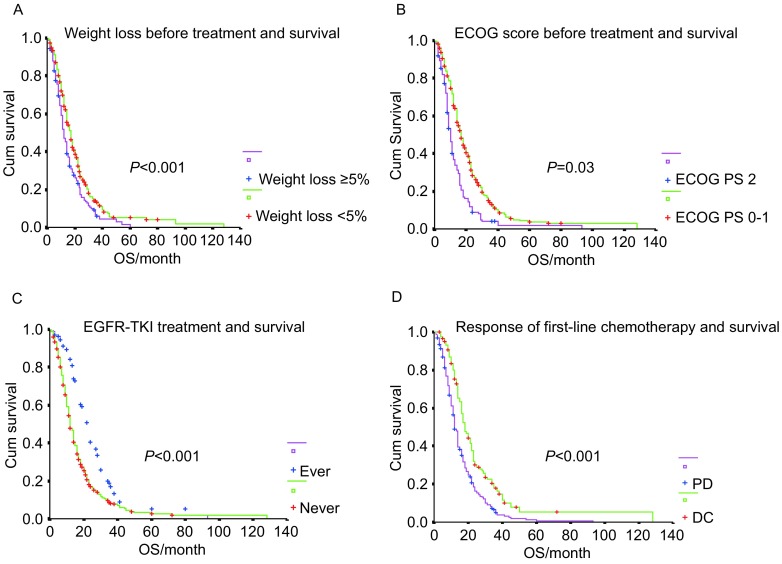
女性晚期非小细胞肺癌患者疗前体重下降（A）、ECOG评分（B）、靶向治疗（C）及一线化疗疗效（D）与生存关系的多因素分析结果。OS：总生存；PD：疾病进展；DC：疾病控制 Multivariate analysis shows weight loss before treatment (A), ECOG score (B), EGFR-TKI treatment (C) and response of first-line chemotherapy (D) are independent prognostic factors for female patients with advanced non-small cell lung cancer. OS: overall survival; PD: progressive disease; DC: disease control

## 3 讨论

随着世界范围内肺癌发病率的逐年升高，特别是女性肺癌新发病例数的激增，肺癌仍然是目前死亡率最高的恶性肿瘤之一。前瞻性研究已经发现，在年龄、病理类型、分期、吸烟状况和治疗等多种因素中，性别可以作为影响生存的一项独立的预后因素^[6]^。对晚期NSCLC患者，女性也是化疗有效的预测因子^[4]^。而对于中国女性患者，哪些因素可以作为最强有力的预后预测指标，目前尚未有共识。本研究系单中心、回顾性大样本研究，对541例女性晚期NSCLC临床病理资料和预后因素进行较为全面的分析，结果显示80.2%为腺癌，总体中位生存时间15个月，1年、2年和5年生存率分别为58.8%、23.7%和3.2%。单因素和多因素分析显示疗前体重下降、ECOG评分、靶向治疗及一线化疗有效为生存的独立预后因素。

女性肺癌患者组织学类型腺癌比例更高，文献^[7]^报道东方女性为63%（*P*=0.003），本研究中高达80.2%，可能与中国女性患者的低主动吸烟率，同时很难避免被动吸烟和环境因素，如大气污染、烹调油烟吸入等有关。目前研究已经证明腺癌患者*EGFR*突变率高达约40%左右^[8]^，该突变是化疗或TKI靶向治疗疗效的有力预测指标。特别是东方女性、腺癌、不吸烟者作为TKI的优势人群，随着靶向药物的应用，女性晚期NSCLC患者的总生存较男性进一步延长。本研究中单因素分析结果，并未得到腺癌与鳞癌相比的生存优势，可能与目前患者的TKI应用比例不高（本研究中约为30%）有关。随着*EGFR*突变检测手段的不断完善，通过分子标志物的检测进一步甄别出有效率高的患者，将性别优势与组织和分子基础相结合，是正确评价女性NSCLC患者预后因素的关键。

目前公认的肺癌预后因素包括疾病分期、PS评分以及确诊前3个月-6个月的体重下降^[4]^。本组无论单因素还是多因素回归分析均显示ECOG评分及疗前体重下降为独立预后因素，表明此组数据具有较好的可靠性和准确性。本研究中Ⅳ期患者共480例，其中Ⅳa期65例，双肺内/远处血行转移者415例，单因素分析二者中位OS和年生存率有明显的统计学差异。2009年国际肺癌研究学会推荐的TNM分期系统将既往划归为湿性Ⅲb期的癌性胸水患者升期至Ⅳa期，主要是基于大样本数据分析显示此亚期患者预后差于真正的Ⅲb期，而接近Ⅳ期。然相较有远处血行转移的Ⅳb期，Ⅳa期是否尚存一定的生存优势仍有待证实。本组中二者中位生存期相差8个月，尽管多因素分析中Ⅳa期并未被证实为独立的预后因素。

接受过EGFR-TKI靶向治疗的患者生存期更长，可能与本组女性患者二、三线选择靶向药物的有效率更高有关。有研究显示女性、腺癌患者接受EGFR-TKI作为二、三线治疗具有更高的有效率和生存时间^[9-11]^。IDEAL1和2研究证实吉非替尼能够改善以前接受过一线或二线化疗的女性晚期NSCLC患者的预后^[10, 11]^。IDEAL2研究中有50%女性患者症状改善，82%达部分缓解，而男性症状改善只占31%^[11]^。INTEREST研究亦提示腺癌和*EGFR*突变者吉非替尼的无病进展生存时间优于多西紫杉醇^[12]^。可能的原因是女性腺癌患者具有更高的*EGFR*突变率，而*EGFR*突变不仅是EGFR-TKI的预测因素亦是预后因素^[13, 14]^。

本研究还观察到，一线化疗疗效是女性晚期NSCLC患者的独立预后因素，一线化疗达到疾病控制的患者中位OS延长6个月，而接受过2个以上二线后化疗方案的患者预后更好，中位OS延长8个月，差别均具有统计学意义。该结果表明一线治疗无效的患者后续治疗有效的概率低、预后差。本研究中还观察到一线化疗疗效对后续治疗及生存时间的影响，这是近年多组回顾性与前瞻性研究关注的重点。如著名的BR21^[15]^和ISEL研究^[16]^，均为探讨厄罗替尼或吉非替尼与安慰剂比较二线治疗晚期NSCLC的国际多中心临床试验，研究设计相似，但结果却迥然相异。BR21呈阳性结果而ISEL研究未发现吉非替尼二、三线治疗优于安慰剂组。其原因之一即二者基线条件不一，ISEL研究入组患者90%左右为难治性NSCLC，而BR21研究中前期化疗失败者仅约40%。Weiss等^[17]^分析了培美曲塞比较多西紫杉醇二线治疗晚期NSCLC的Ⅲ期随机多中心临床研究中一线疗效对二线治疗的影响，结果显示性别、分期、PS评分和一线化疗有效对总的中位生存时间有明显影响，尤其一线化疗达有效者，OS获明显改善。这一研究结果的深层原因是否与药物敏感和耐药有关尚待研究，但至少提示我们今后设计新的二线治疗临床研究时，一线治疗疗效应该作为重要的分层因素。

综上，在女性肺癌预后相对较好的背景下，通过临床观察发现有力的预后预测因素，并深入探讨其潜在的分子机制，将有利于真正改善女性晚期NSCLC患者的生存，提高肺癌的整体诊治水平。
